# Opportunistic Identification of Vertebral Compression Fractures on CT Scans of the Chest and Abdomen, Using an AI Algorithm, in a Real-Life Setting

**DOI:** 10.1007/s00223-024-01196-2

**Published:** 2024-03-26

**Authors:** Magnus Grønlund Bendtsen, Mette Friberg Hitz

**Affiliations:** 1 Research Unit, Medical Department, Zealand University Hospital, Koege, Denmark; 2 Institute of Clinical Medicine, University of Copenhagen, Koege, Denmark

**Keywords:** Osteoporosis, Vertebral fracture, Fracture prevention, Health services research, Osteoporosis, Radiology, Screening

## Abstract

This study evaluated the performance of a vertebral fracture detection algorithm (HealthVCF) in a real-life setting and assessed the impact on treatment and diagnostic workflow. HealthVCF was used to identify moderate and severe vertebral compression fractures (VCF) at a Danish hospital. Around 10,000 CT scans were processed by the HealthVCF and CT scans positive for VCF formed both the baseline and 6-months follow-up cohort. To determine performance of the algorithm 1000 CT scans were evaluated by specialized radiographers to determine performance of the algorithm. Sensitivity was 0.68 (CI 0.581–0.776) and specificity 0.91 (CI 0.89–0.928). At 6-months follow-up, 18% of the 538 patients in the retrospective cohort were dead, 78 patients had been referred for a DXA scan, while 25 patients had been diagnosed with osteoporosis. A higher mortality rate was seen in patients not known with osteoporosis at baseline compared to patients known with osteoporosis at baseline, 12.8% versus 22.6% (p = 0.003). Patients receiving bisphosphonates had a lower mortality rate (9.6%) compared to the rest of the population (20.9%) (p = 0.003). HealthVCF demonstrated a poorer performance than expected, and the tested version is not generalizable to the Danish population. Based on its specificity, the HealthVCF can be used as a tool to prioritize resources in opportunistic identification of VCF’s. Implementing such a tool on its own only resulted in a small number of new diagnoses of osteoporosis and referrals to DXA scans during a 6-month follow-up period. To increase efficiency, the HealthVCF should be integrated with Fracture Liaison Services (FLS).

**Supplementary Information:**

The online version contains supplementary material available at 10.1007/s00223-024-01196-2.

## Introduction

Osteoporosis is a systemic bone disease characterized by reduced bone mass and micro architectural deterioration of bone tissue, increasing the risk of fractures [1]. Fragility fractures, especially vertebral compression fractures (VCF), are a significant health concern associated with higher morbidity [2–5] and mortality [6, 7]. The importance of prevalent vertebral deformities has also been investigated, indicating that vertebral deformity is correlated with risk of all fractures. This underscores the significance of early VCF detection to initiate osteoporosis treatment for fracture prevention [8–10]

Unlike other osteoporotic fractures, VCFs often go unnoticed due to various factors, including the absence of symptoms, non-specific symptoms, and limited clinical attention [11–13]. VCFs not only increase the imminent fracture risk but also the lifetime risk of fractures [14–16].

In the clinical context, the definition of a VCF remains uncertain. Various methods, including semi-quantitative, quantitative or a combination of both, are used to evaluate vertebral body height and shape, although a gold standard does not exist [17]. The semi-quantitative method described by Genant is easier to implement with a fair degree of reproducibility and accuracy. Studies have shown moderate to good correlation between Genant’s semiquantitative and quantitative methods, especially for moderate to severe fractures [17].

VCFs can be diagnosed incidentally during computed tomography scans (CT scans) ordered for reasons unrelated to osteoporosis. However, these fractures are often not reported in radiology reports because their clinical importance is not adequately emphasized. Identifying VCFs opportunistically during CT scans for any indication could improve patient identification without significantly burdening hospitals in terms of time and resources [18–20]. Thus, it provides the clinician with the opportunity to treat the patient and reduce future fracture risk. To address this issue, Nano-X Ai Ltd has developed the HealthVCF algorithm, aimed at assisting radiologists in detecting frequently overlooked lesions. The HealthVCF is a passive notification for prioritization-only, parallel-workflow software tool used by clinicians to prioritize specific patients within the standard-of-care bone health setting for suspected vertebral compression fractures. It uses an artificial intelligence algorithm to analyze chest and abdominal CT scans and flags those that are suggestive of the presence of at least one vertebral compression at the exam level. The operational performance of the HealthVCF has been examined previously. In a study conducted at an Israeli hospital, both training and validation were performed within the same study. Validation was conducted on 15% of 1673 CT scans of the chest or abdomen from 3701 adults aged 50 or older, comprising approximately 250 CT studies. The algorithm demonstrated a sensitivity of 84% and a specificity of 94% [21]. In a US hospital, the algorithm showed sensitivity and specificity in diagnosing any VCF of 0.66 and 0.90, respectively. For diagnosing moderate/severe VCF the sensitivity and specificity were 0.78 and 0.87 [22], respectively. A study conducted in Australia investigated the HealthVCF in a real-life clinical setting at a single imaging center, independent of the software’s development/training site. Here, the detection of Genant 2 or Genant 3 fractures showed sensitivity of 65%, specificity of 92%, and accuracy of 88% in 1288 scans [23]. The poorer performance in the Australian study is an example of limited generalizability, where AI algorithms show great performance in development environments, but poorer performance when used on real-world data [24]. To assess the real-world performance of an AI algorithm such as the HealthVCF, a paired design can be used to do a comparative evaluation of the performance between the AI algorithm and the conventional way of diagnosing vertebral fractures [24].

The aim of our study was to assess the performance of an updated version of the algorithm in identifying prevalent moderate/severe VCFs on CT scans of thorax and abdomen (CTAB) in a real-life setting and to evaluate the impact of implementation on registration of diagnoses, referral to DXA and medical treatment.

## Methods

### Study Design

The HealthVCF, version 5.1.1, was installed and configured for the highest specificity on a server in January 2021 at the Department of Radiology, Zealand University Hospital (ZUH). It was set to identify Genant 2–3 (moderate/severe) VFCs on CTAB of patients referred to the Radiology Department during the study period, provided patients met the inclusion criteria. No human interaction was involved in handling of the input data.

If a moderate/severe VCF was identified by the HealthVCF, an additional scan image was added by the software to the CT scan series, before the examination was returned to the imaging system (Radiology Information system), RIS (Picture Archiving and Communication System, PACS). The HealthVCF used the Genant semi-quantitative method for diagnosing VCFs. The evaluation by the HealthVCF was done in real-time and did not delay the conduct of the CTAB. Scans identified with one or more moderate/severe compression fractures, were denoted positive scans. All scans evaluated by the HealthVCF were identified by accession number, registered on a weekly worklist with indications of the presence of moderate/severe VCF, and sent to the study team.

Following standard procedures all scans were evaluated in real-time by the department’s radiologists and specialized radiographers according to clinical question. A report of evaluation was sent to the referring physician according to standard procedures.

A group of five specialized radiographers, with an average of 21 years (10–28 years) of radiographic experience including evaluation of CT scans of the spine, constituted the study team. Their instruction was to evaluate whether one or more moderate/severe VFCs were present. The study team was trained in the workflow prior to the start of study, and in inconclusive cases a senior radiologist was consulted. The evaluation by the specialized radiographers and/or radiologists constituted the gold standard.

#### Validation Study

To evaluate the performance of the algorithm in a Danish population, a total of 1000 CTAB were randomly selected and evaluated by both specialized radiographers and the HealthVCF. The radiographers were blinded to both the study report and the outcome from the HealthVCF, when evaluating the scans. The 1000 CTAB were selected by using Microsoft Excel to randomly assign each scan from the study period with a number and then present scan 1 to 1000 on a worklist for evaluation by the study team. No reporting of findings was done in this part of the study.

#### Follow-Up Cohort at 6 Months

After a 6-month follow-up period, a retrospective cohort was formed. The cohort was formed by having the study team analyze all scans flagged positive for a VCF by the HealthVCF. Only patients with CTAB determined true positive for a VCF were included in the cohort. CTABs not flagged by the HealthVCF were not analyzed by the study-team but were analyzed by the radiologist at the time of CTAB, following standard procedure. For CTAB determined true positive for a VCF by the study team, the radiographer looked up the initial report. If no VCF had been reported, a standardized sentence was added to report alerting the referring physician to the VCF and noting that osteoporosis assessment was advised. If the VCF was already described in the initial report, no action was taken. After 6 months from CTAB all data were entered into a Redcap database.

### Study Population

The study population includes both inpatients and outpatients at Zealand University Hospital in Koege referred for CTAB for all indications except fractures. The study included both women and men, aged 50 years and older. Individuals without a social security number (tourist) and individuals from other geographical regions were excluded. The aim was to evaluate 10,000 CTABs, and the study period ended when this number was reached. The cohort identification period was from 01.23.2022 to 10.25.2022.

Exclusion criteria encompassed cases involving CTAB conducted as part of PET scans and instances where assessment of the vertebral column was not possible as judged by the radiographer/radiologist. CTAB for fracture identification were excluded, as well as cases of incomplete exams and wrong region of interest. No performance error analysis was conducted as it was outside the study’s scope.

The study was approved as a quality assurance/development study by the local data committee (REG-145-2021) and the hospital’s board of directors, at the guidance of the Danish Health Authorities. Approval by the ethical committee was not required for this type of study, as no intervention was done, and all patient-related data was collected retrospectively. Adherence to CONSORT-AI 2020 guidelines is detailed in the supplementary material.

### Variables: Follow-Up Cohort

At the time of the CTAB (baseline), the following data were registered for each patient: age, sex, indication for CTAB, specialty of referring physician, known with osteoporosis (yes/no), current treatment for osteoporosis (yes/no), medication, and for description of work-flow, whether the fracture was described in the primary radiology report or added by study team.

At 6 months follow-up the following data were registered for each patient: vital status, referral for DXA scan, specialty of referring physician, initiation or adjustment of osteoporosis treatment, choice of treatment, and registration of the osteoporosis diagnosis.

Patients were classified as having known osteoporosis if any of the following criteria were met: a diagnosis code for osteoporosis in the electronic patient journal (EPJ), a DXA scan result with a T-score below or equal to − 2.5 in lumbar spine or hip region, or if the patient was receiving anti-osteoporosis medication.

Details on indication for CTAB, specialties of referring physicians and data sources are found in supplementary material.

### Statistics

To assess the test’s diagnostic accuracy, the number of true positives, false positives, true negatives, and false negatives was calculated. Sensitivity, specificity, positive and negative likelihood ratios, Youden Index, as well as positive and negative predictive values were calculated by means of standard formulae. A joint 95% confidence interval was calculated using Clopper-Pearson formula.

Descriptive data were presented as number of patients in each category; groups were compared using either independent sample t-test for continuous data or Pearsons Chi-square test for categorical data. If necessary, p-values were Bonferroni corrected by multiplying p value with n = test for n ≤ 6.

Statistical significance of 5% was used. All data were analyzed with SPSS (SPSS Inc, Chicago, Illinois), version 21.

## Results

### Validation Study and Identification of Retrospective Cohort

One thousand scans were evaluated in the validation study, with sensitivity being 0.68 (CI 0.58–0.78) and specificity 0.91 (CI 0.89–0.93) (Table 1). Accuracy was 88.9% and Youden Index was 0.59. The prevalence of one or more moderate or severe VCFs for the population in the validation study was 9.5%.

**Table 1:** Performance analysis of the algorithm in a Danish population of 1,000 patients

	Evaluation by algorithm		
	Zebra positive	Zebra negative	Total
Evaluation by radiographers			
VCFs confirmed	65	30	95
No VCFs	81	824	905
Total	146	854	1000

Analysis	Results (CI)
Sensitivity	0.684 (CI 0.581;0.776)
Specificity	0.910 (CI 0.890;0.928)
Positive predictive value	0.445 (CI 0.363;0.530)
Negative predictive value	0.965 (CI 0.950;0.976)
Positive likelihood ratio	7.65 (CI 5.96–9.80)
Negative likelihood ratio	0.347 (0.258–0.467)

CI were calculated using Clopper–Pearson formula (mean ± 1.96 SD)

*CI* confidence intervals, *VCF* vertebral compression fracture

**Fig. 1:**
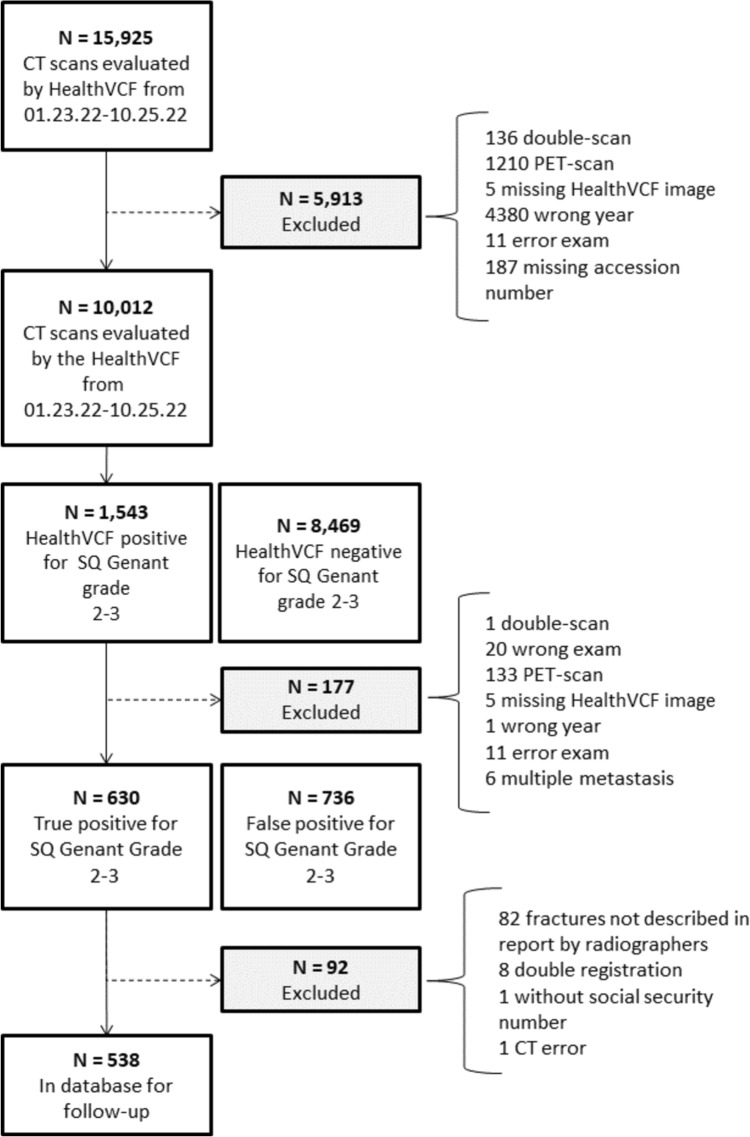
Flow-chart of CT scans evaluated during the cohort identification period

During the cohort identification period, a total of 10,012 CTABs were included. Among these, 1543 CTABs were categorized as HealthVCF positive for moderate/severe compression fractures. Evaluation of the HealthVCF-positive scans resulted in exclusion of 177 scans. Of the remaining 1366 HealthVCF-positive scans, 630 were found to be true positive for moderate to severe VCFs. Of the 630 CT scans, 538 were included in the database as the retrospective cohort (Fig. 1). Eighty-two of the excluded scans were excluded due to absence of documentation on fractures in the radiology reports. The lack of documentation resulted from the evaluator’s judgment that the subjects had a limited life expectancy, constituting a breach of the protocol. The final retrospective cohort consisted of 318 women and 220 men.

Mean age of participants was 76 ± 8 years, and women were significantly older than men, 77 ± 8 years versus 75 ± 8 years (p = 0.013). At the time of the CTAB (baseline), a total of 250 patients (46.4%) were diagnosed with osteoporosis. Among them, a significantly higher percentage were women (74.4%) (p < 0.0001) (Table 2).

**Table 2:** Characteristics of patients and baseline data for baseline cohort, n = 538

	Patients
Total	538
Age, mean (SD)	76 (8)
F/M ratio	318/220
OP diagnosis, n	250
No OP diagnosis, n	288
Women	318
Age, mean (SD)	77 (8)
OP diagnosis, n	186
No OP diagnosis, n	132
Men	220
Age, mean (SD)	75 (8)
OP diagnosis, n	65
No OP diagnosis, n	156
Medical treatment in diagnosed patients (n = 250)	
Yes, n (%)	171 (68.4)
No, n (%)	79 (31.6)
Indication for scan (n = 538)	
Acute indication	
n	200
Age, mean (SD)	77 (9)
F/M ratio	117/83*
Diagnostic exam	
n	117
Age, mean (SD)	76 (8)
F/M ratio	60/57**
Oncological exam	
n	127
Age, mean (SD)	76 (8)
F/M ratio	92/35
Control scan, other	
n	94
Age, mean (SD)	77 (7)
F/M ratio	49/45***

The proportion of women compared to men was significantly higher for the oncological exam compared to acute exam (p < 0.03)*, diagnostic exam (p < 0.003)** and control scan (p < 0.006)***. The p-values were Bonferroni corrected. No difference was found in female male ratio between the other indications

*SD* standard deviation, *F/M* female/male, *n* number of patients

Of the patients classified as having known osteoporosis at baseline, 68.4% were receiving anti-osteoporosis treatment, 44.4% had a DXA scan with T-score < − 2.5 in either the hip region or lumbar spine and 52.0% had a registered diagnose of osteoporosis (see supplemental material). The distribution of anti-osteoporosis treatment in the 171 patients (68.4%) receiving anti-osteoporosis treatment at baseline is shown in Table 3.

**Table 3:** Osteoporosis diagnoses, deaths and medication in baseline cohort, n = 538 and 6 months follow-up cohort, n = 441

	Patients		
	Known with OP at baseline	Not known with OP at baseline	Total
Baseline cohort, n	250	288	538
Anti-osteoporosis treatment at baseline, n	171	0	171
Oral bisphosphonates	90		
I.V bisphosphonates	45		
Denosumab	35		
Teriparatide	1		
Romosozumab	0		
Unknown	0		
Deaths during follow-up, n	32	65	97
6 months follow-up cohort, n	218	223	441
Anti-osteoporosis treatment at baseline, n	154	0	154
Oral bisphosphonates	83		83
I.V bisphosphonates	39		39
Denosumab	31		31
Teriparatide	1		1
Romosozumab	0		0
Unknown	0		0
No anti-osteoporosis treatment at baseline, n	64	223	287
Anti-osteoporosis treatment at 6 months follow-up, n (changes from baseline)	154	23	177 (+ 23)
Oral bisphosphonates	68	14	82 (− 1)
I.V bisphosphonates	40	5	45 (+ 6)
Denosumab	37	0	37 (+ 6)
Teriparatide	3	2	5 (+ 4)
Romosozumab	0	2	2 (+ 2)
Unknown	6*	0	6 (+ 6)
No anti-osteoporosis treatment at 6 months follow-up, n (changes from baseline)	53	200	253 (− 34)
Referred for DXA at 6 months follow-up, n	41	37	78

*OP* osteoporosis

*Under medical treatment revision—new treatment to be decided

Indication for CT scan is seen in Table 2, and the specialty of referring physician is shown in the supplementary material.

For work-flow analysis, the findings of a VCF were described in the primary radiology report in 91% of cases and added secondarily in 9% of the cases. More VCFs were described in the primary report towards the end of the study period, indicating a time factor. In the first half of the study, 12.9–26.5% of the findings were added after the primary report, while in the second half, this occurred in only 0.0–4.2% of the cases (p < 0.001).

### 6-Months Follow Up

At 6-months follow up, 441 of the patients in the retrospective cohort were alive, 97 had died, and none were lost to follow-up (Table 3). Significantly more patients not known with osteoporosis at baseline were dead at 6 months compared to patients known with osteoporosis at baseline; 12.8% versus 22.6% (p = 0.003). There was no difference in mortality between men (20.9%) and women (16.0%) (p = 0.148), and no difference in mortality between the retrospective cohort (18.0%) and the 82 patients excluded from the cohort by radiographers’ choice not to evaluate the CTAB (22.8%) (p = 0.276). Patients receiving bisphosphonates had a lower mortality of 9.6% compared to the rest of the population 20.9% (p = 0.003).

**Fig. 2:**
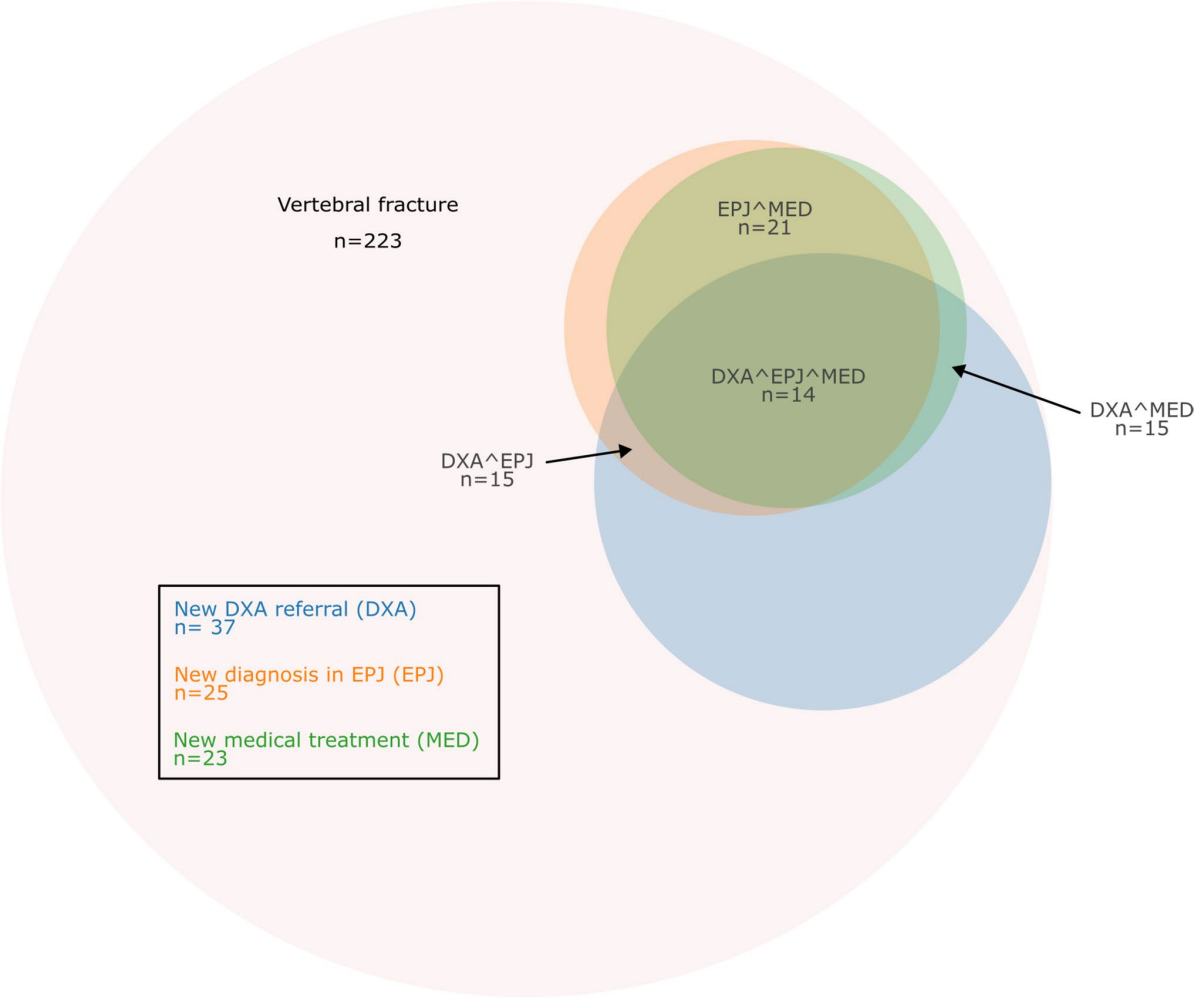
Diagnostic workflow upon CT verified vertebral fracture in population not known with osteoporosis at baseline, n = 223. *N* number of patients, *DXA* dual-energy X-ray absorptiometry, *EPJ* electronic patient journal

The mortality in the retrospective cohort, stratified by the indication for the CTAB, was as follows: 62/200 (31.0%) for acute exams, 12/117 (10.3%) for diagnostic exams, 20/127 (15.7%) for oncological control exams and 3/94 (3.2%) for other control exams. The mortality was significantly higher for patients referred for an acute exam compared to diagnostic exam (p = 0.006) and other control scans (p = 0.006) but not significantly higher compared to patients referred for an oncological exam (p = 0.12) after Bonferroni correction. Furthermore, mortality of patients referred for other control exams was lower compared to patients referred for oncological exam (p = 0.018) but not when comparing to patients referred for a diagnostic CT scan (p = 0.282) after Bonferroni correction. Among the 441 patients in the follow-up cohort, 218 were known with osteoporosis at baseline. At 6 months follow up, 37 of the 223 patients not known with osteoporosis at baseline were referred for DXA scan, 25 had a new diagnosis of osteoporosis in EPJ, and 23 were started on anti-osteoporosis treatment (Fig. 2).

Of the 218 known with osteoporosis at baseline, 154 were on medical treatment at baseline (Table 3). Out of the 64 patients not on medical treatment, 41 were referred for DXA, and 11 were started on anti-osteoporosis treatment at the time of the 6-months follow-up. None were registered with a new diagnosis in EPJ based on the result. Of the 154 patients on anti-osteoporosis treatment at baseline 16 were changed in medication based on the results (see supplemental material).

### DXA Referral and Workflow

In the follow-up cohort a total of 78 were referred for DXA scan. There was no difference in age 74.9 years (8.9) and 76.4 (7.7) [mean (SD)] (p = 0.140) or female/male ratio (48/30) and (219/144) (p = 0.843), for those referred for DXA versus those who were not, respectively.

Out of the 78 patients referred for a DXA scan, 32.1% (n = 25) were referred by the same physician who did the initial referral to the CT scan. Among these, 40.0% (n = 10) came from the medical department, 36% (n = 9) from general practitioners (GP), and 8.0% (n = 2) from the surgical department and private surgical clinics respectively. Additionally, 4.0% (n = 1) were referred by the intensive care unit and the oncological department, respectively.

Fifty-three (67.9%) patients were referred for a DXA scan by other physicians, with 43.4% (n = 23) coming from the medical department, 35.8% (n = 19) from general practitioners, 13.2% (n = 7) from specialist clinics, and 1.9% (n = 1) from the intensive care unit and the emergency department, respectively.

## Discussion

### Performance of the Algorithm and Fracture Prevalence

Our objective was to validate the HealthVCF algorithm within a Danish hospital environment and to explore the impact of its implementation on osteoporosis diagnosis and management over a 6-month follow-up period. The sensitivity for finding moderate/severe VCFs were 68%, and the specificity 91%. This is, especially for the sensitivity, below what is shown in the FDA approval of the HealthVCF, where sensitivity and specificity were 90.20% (95% CI [86.35%;93.05%]) and 86.89% (95% CI [82.63%;90.22%]), respectively.

A recent meta-analysis comparing the performance of similar algorithms showed better average performance across 14 algorithms, with sensitivity 85.7% (95% CI 78.6–90.7) and specificity 93.5% (95% CI 89.5–96.1) [25]. Comparing our results to the results of the meta-analysis suggests that the tested HealthVCF has poorer performance than what could be expected.

Comparing our results with results of Kolanu et al. [23], who tested the same algorithm, we found an almost identical performance. Together with results from Australia, our results point to a poorer performance of the HealthVCF than what is shown in the regulatory data. The subpar performance is a testament to the lack of generalizability to the Danish population.

The accuracy of the HealthVCF was 88.9%, and it demonstrated a Youden Index of 0.59.

A Youden Index of 0 is equivalent to a coin toss, and a Youden Index of 1.0 represents the perfect test. With a Youden Index of 0.59, the HealthVCF has a fairly good performance. To determine its usefulness, sensitivity and specificity must be considered. With a sensitivity of 0.68, approximately 32% out of all scans flagged positive for vertebral fracture will be false positive. With such a low sensitivity, the HealthVCF cannot be used to diagnose vertebral fractures without human oversight, as stated by the manufacturer in their FDA application. This over-calling of fractures means that all positive scans must be analyzed by a specialist, leading to increased expenditure. In our study radiographers spent 5–10 min on average analyzing each image generated by the HealthVCF, including lookup, analysis, and potential reporting. In 1000 scans, this translates to somewhere between 12 and 24 h of work by radiographers to correctly diagnose fractures in 65 patients out of 146 flagged scans.

Focusing on the specificity of 0.91, things are looking better. About 9 out of 10 scans not flagged by the HealthVCF will be true negative. This means that the radiographic specialist can be fairly sure that no vertebral fracture is present when a scan is not flagged by HealthVCF. Though this is not perfect, the HealthVCF might be used by a Department of Radiology to increase their reporting of vertebral fractures without the need to analyze the spine of every CTAB. This minimizes the resources spent on opportunistic identification of vertebral fractures.

The variability in performance compared to previous studies may be explained by differences in population fracture risk and the variability in evaluation based on the method of evaluation [17, 21]. Morphometric evaluation methods, whether quantitative or semi-quantitative have been thoroughly documented [17, 26] and diagnosing Grade 1 (mild) VCFs presents a diagnostic challenge due to notable discrepancies in interpretation among radiologists, leading to an elevated risk of false positive results.

Both clinical and non-clinical VCFs are associated with future fractures risk with lower association for grade 1 compared to grade 2 and 3. This might be explained by the lower specificity of the methods in diagnosing mild fractures [8, 10, 27–29]. Based on this knowledge, we focused on identifying only moderate/severe VCFs in this study.

The prevalence of patients with moderate/severe VCFs in the validation study was 9.5%. This is a low prevalence compared to previous studies. The prevalence of VCFs varies in different studies and populations, which may in part be explained by difference in population-risk, method used for evaluation, but also the grades of fractures included in the studies. One study reported the prevalence of morphometric VCFs in Scandinavia to be 26% and 18% in Eastern Europe [30]. Another study found a prevalence of 12% [31] and a third a prevalence of 9.5% [20]. Though age ranged from 20 to 88 years in the third study, no VCFs were identified in the younger patients (< 44 years). A different study found an overall prevalence of 35%; 73% of these were grade 1 fractures, 19% grade 2 and 9% grade 3 [18]. The prevalence for moderate/severe VCFs in the study was 9.5%, equivalent to our report, indication that that part of the variability in prevalence between studies is related to the diagnosis of mild fractures.

In our study, 48% of patients in the retrospective cohort had known osteoporosis at baseline; of these 74.4% were women. This is anticipated due to the higher incidence of osteoporosis in women and the tendency for osteoporosis in men to go unnoticed [32, 33]. Many studies have evaluated incident findings of VCF on chest radiographs or CTAB, but few studies report on patients’ osteoporosis- and treatment status at the time of identification. Barton et al. evaluated the clinical outcome of VCF identification and reported that at time of VCF diagnosis, 21% were receiving anti-osteoporosis treatment [34]. In comparison, we found that 31% of the retrospective cohort were receiving treatment at baseline. Patients in this study were labeled known with osteoporosis based on either an osteoporosis diagnose code in the EPJ, DXA results with a diagnostic T-score in the EPJ or current treatment with anti-osteoporosis drug. This method may lack precision due the existence of different EPJ systems in Denmark. If patients have previously resided in other geographical regions, their DXA results may go unnoticed. Adding to this, patients diagnosed at their GP may not have a diagnosis in the EPJ, resulting in an underestimation of patients with an osteoporosis diagnosis. Conversely, information about current treatment and diagnosis is nationally centralized. Our analysis revealed that 48% of patients with known osteoporosis did not possess an official diagnosis but were either undergoing treatment or had a diagnostic T-score. To be able to provide an accurate representation of disease prevalence, proper diagnosis registration is essential.

### Effect of Intervention

At the 6-months follow-up, 97 of the patients in the retrospective cohort had died, resulting in a mortality rate of 18%. The mortality was higher in the group of patients not known with osteoporosis (68%) compared to the group known with osteoporosis (32%). This was not explained by differences in reason for referral (indication for CT scan). Comorbidity, like cancer, and previous osteoporotic fractures, especially hip fractures, may be confounding factors explaining the difference in mortality between the two groups, but we have no data on these variables. Bisphosphonates were used for treatment in 79% of the patients under current osteoporosis treatment in the cohort. The mortality in patients treated with bisphosphonates was significantly lower compared to the rest of the cohort, i.e. patient on other treatments or not in current treatment. Bisphosphonates were only registered if the indication was osteoporosis. Other studies have shown that treatment with bisphosphonates reduces overall mortality, but there is not sufficient data to support this hypothesis [35–38]. Another explanation for a higher mortality in the group not known with osteoporosis, may be lack of attention toward osteoporosis in presence of other chronic- or severe disease. It is known that a large treatment gap is present in osteoporosis, and it is assumed that the gap is linked to both a gap in diagnosis and lack of awareness. It is not known if the presence of other chronic or severe diseases is the cause of the gap in osteoporosis diagnosis [39].

The aim of opportunistic identification of VCFs is to ensure appropriate assessment and treatment of the patients to reduce risk of future fractures and mortality. At 6 months, 18% of the retrospective cohort were referred for a DXA scan and 11% were either started on anti-osteoporosis treatment or changed to a more potent anti-osteoporosis treatment. As noted earlier, few studies report on clinical outcomes of fracture identification, but Barton et al. reported that within 2 years after a clinical VCF, 2% of patients had a DXA scan and 7% initiated anti-osteoporosis treatment [34]. Thus, our results show a higher DXA referral rate compared to the study by Barton et al.

### Workflow of Action

Analysis of the workflow data showed that GPs and medical specialists had the highest rate of acting on the reporting of a VCF. This can be due to both GPs and medical specialists being specialties with knowledge on diagnosis and treatment of osteoporosis.

The analysis also showed that 91% VFC’s detected were described in the primary radiology report, and that the highest rate was seen at the end of the study. This indicates increased awareness among radiologists during the study, but it also demonstrates that a high rate of VCF detection is only part of the solution to closing the treatment gap, given that only 25 of the 233 patients not known with osteoporosis received an osteoporosis diagnosis within 6 months. Higher efficiency could be accomplished by integrating HealthVCF with a fracture prevention program like Fracture Liaison Service (FLS), an internationally validated method of systematically identifying, evaluating and initiating anti-osteoporosis treatment for patients with recent fractures [40]. The programs are shown to increase likelihood for subsequent BMD testing [41, 42].

The rate of VCF reporting in this study is higher than in the literature, where the reporting of incident VCFs on CT scans was found to be 9% [18], 13% [19], 14.6% [20], 32.56% [43] and 24.7% [13]. The high report rate in our study is likely due to the radiologists’ knowledge of the ongoing study, whereas the underreporting noted in other studies might be due to radiologists’ lack of focus on bone-status, as opportunistic screening for VCFs is a non-acute finding during exams often performed in an acute setting [44]. The problem of underreporting might be solved by using a dedicated team to evaluate the HealthVCF findings outside the clinical context, as done in this study. With such a workflow, the radiologists sparse time and resources are preserved for more acute findings.

In this study, 46.7% of the deaths that occurred during the 6-month follow-up period had an acute exam or an oncological follow-up as indication for the CTAB. This shows that opportunistic screening might be inefficient in this subset of the population. Additionally, we observed that the radiographers opted to exclude certain patients from the cohort due to their assessment of a short life expectancy. However, the mortality in this group was no different from that of the rest of the cohort, thus demonstrating that it is difficult to predict mortality. The high mortality rate of 18% in the studied population underscores the need to incorporate a way to spare the system of ineffective use of resources and to spare very ill patients the time and energy of attending consultations, going to scans and initiating unnecessary treatment. This could be done by developing a clinical decision tool to help the FLS team to identify which patients to refer for further assessment and treatment based on their risk of dying and the predicted benefit of attending an FLS. Such a tool has previously been developed by Ong et al. [45].

### Strengths and Limitations

One limitation of this study is the choice to use only one radiographer instead of two to determine the presence of fractures in each of the 1000 scans used for evaluation of performance. Ideally two radiographers would conduct a separate analysis of each scan to ensure validity, though the use of only one radiographer does not seem to have affected the result, as our sensitivity and specificity are similar to that found in a recent study of the same algorithm [23]. Another limitation is the short follow-up period, as it might be too limited a time frame for every physician to act on the radiologist’s report, especially in the presence of other severe diseases. Finally, the number of patients diagnosed with osteoporosis during the follow-up period might be underestimated due to osteoporosis diagnoses not being registered in the EPJ when given by a GP.

A strength of this study is the use of a retrospective cohort, giving data on indication for scan, diagnosis, medications and referral to DXA. This data is what makes it possible to determine the real-life impact of an algorithm such as the HealthVCF, as the end goal is not only to detect but to prevent fractures.

## Conclusion

In conclusion we confirm previous findings of the HealthVCFs poor performance in a real-world setting. It is not generalizable to the Danish population as it is over-calling patients and missing 1 in 10 VCFs. Even though the tested version of the HealthVCF needs improvement in accuracy before it can be used for opportunistic identification of VCF independently of human oversight, the current version can be used to increase reporting of vertebral fractures by prioritizing which CT scans to analyze for potential VCF. Implementing the HealthVCF with human oversight without ties to FLS, only resulted in a low number of new diagnoses of osteoporosis and referrals to DXA among patients not known with osteoporosis at time of the CT scan. This finding, together with the high mortality rate in the studied population and lack of action among specialties other than GP’s and medical specialists, highlights a need for FLS-teams to ensure the efficiency of the HealthVCF.

### Supplementary Information

Below is the link to the electronic supplementary material.

Supplementary file1 (DOCX 64 kb)

## Data Availability

The AI code is property of Nano-x AI Ltd, and the authors had no access to the code during the study.
