# Supplementary material for: Opportunistic Identification of Vertebral Compression Fractures on CT Scans of the Chest and Abdomen, Using an AI Algorithm, in a Real-Life Setting
Source: Calcif Tissue Int. 2024 Mar 26;114(5):468–79. doi: 10.1007/s00223-024-01196-2 (PMC11061033; doi:10.1007/s00223-024-01196-2)
Supplement: Supplementary file 1 — Supplementary file1 (DOCX 64 kb) [file 223_2024_1196_MOESM1_ESM.docx]

Supplementary Material

Opportunistic identification of vertebral compression fractures on CT scans of the chest and abdomen, using the Zebra Medical Imaging algorithm, in a real-life setting

Calcified Tissue International

Mette Friberg Hitz, Magnus Grønlund Bendtsen

Corresponding author: Magnus Bendtsen, Research Unit, Medical Department Zealand University Hospital, Denmark, magnb@regionsjaelland.dk

Table S1: Indication for scan and referring physician, n= 538

|  |  | Acute indication | Diagnostic exam | Oncological exam | Control scan, other | Total |
| --- | --- | --- | --- | --- | --- | --- |
| Referring specialty, n | |  |  |  |  |  |
|  | Intensive care Unit | 6 | 3 | 0 | 0 | 9 |
|  | Emergency Department | 87 | 1 | 0 | 0 | 88 |
|  | Surgical Departments | 43 | 33 | 6 | 33 | 115 |
|  | Medical Departments | 60 | 31 | 7 | 52 | 150 |
|  | Dermatological Department | 0 | 6 | 4 | 1 | 11 |
|  | General Practice | 3 | 42 | 0 | 4 | 49 |
|  | Private clinic | 1 | 1 | 13 | 2 | 17 |
|  | Oncological Department | 0 | 0 | 97 | 2 | 99 |

^n: number of patients^

Table S2: Basis for osteoporosis diagnosis in the baseline cohort, n=538

|  |  | DXA scan with T-score < -2,5  (hip and/or lumbar spine) | | | Total |
| --- | --- | --- | --- | --- | --- |
|  |  | Yes | No | Unknown |  |
| Diagnosis registered in EPJ* | |  |  |  |  |
|  | Yes | 66 | 32 | 32 | 130 |
|  | No | 45 | 19 | 56 | 120 |
|  | Total | 111 | 51 | 88 | 250 |

^* EPJ = Electronic Patient Journal^

Table S3: Basis for osteoporosis diagnosis in the baseline cohort, n=538

|  |  | Receiving anti-osteoporosis medication | | Total |
| --- | --- | --- | --- | --- |
|  |  | Yes | No |  |
| Diagnosis registered in EPJ | |  |  |  |
|  | Yes | 90 | 40 | 130 |
|  | No | 81 | 39 | 120 |
|  | Total | 171 | 79 | 250 |

^* EPJ = Electronic Patient Journal^

Table S4: Basis for osteoporosis diagnosis in the baseline cohort, n=538

|  |  | Receiving anti-osteoporosis medication | | Total |
| --- | --- | --- | --- | --- |
|  |  | Yes | No |  |
| Diagnostic DXA scan | |  |  |  |
|  | Yes | 90 | 21 | 111 |
|  | No | 24 | 27 | 51 |
|  | Unknown | 57 | 31 | 88 |
|  | Total | 171 | 79 | 250 |

^* EPJ = Electronic Patient Journal^

Fig. S1: Medical treatment at time for CT scan (baseline) and 6 months follow-up


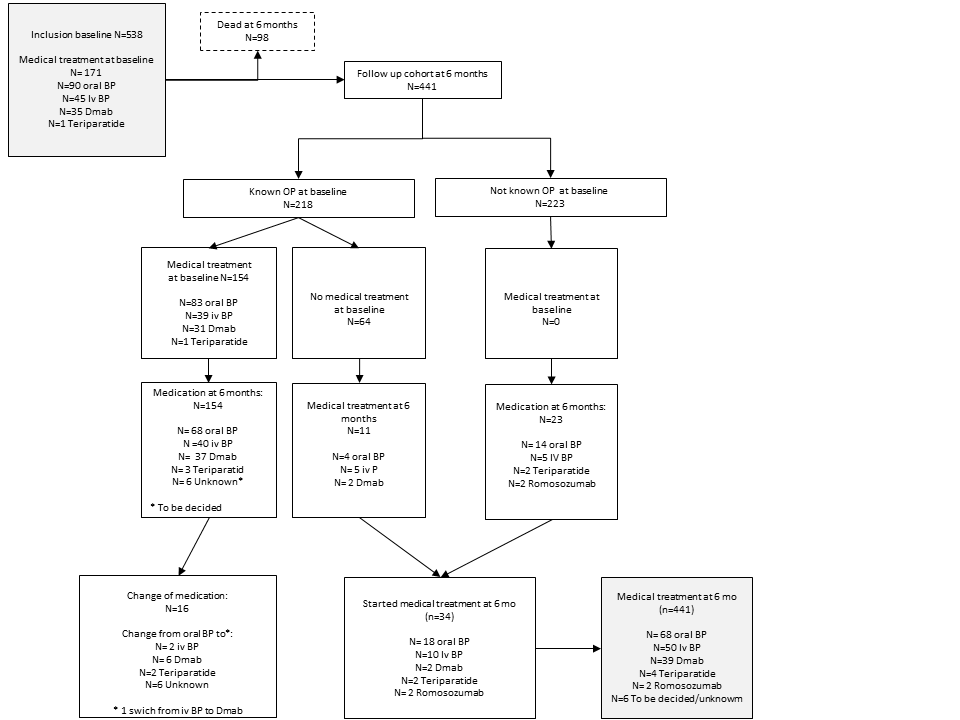


Fig. S2: Osteoporosis diagnoses, registered medical treatment and DXA results at CT scan (baseline)


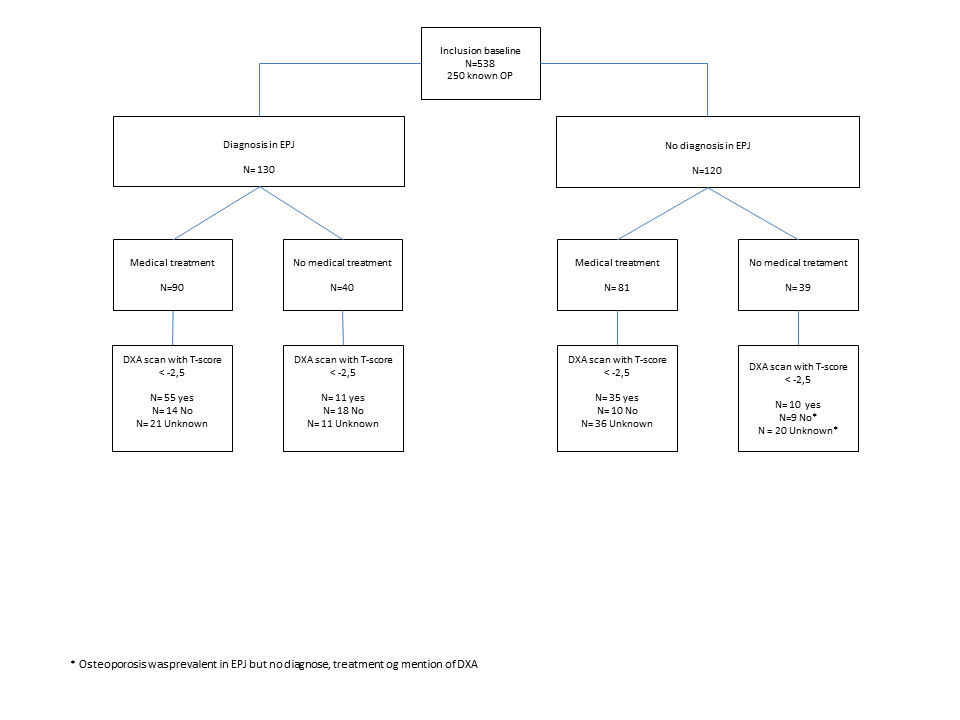


Table S5: Data sources

| **Source** | **Data** |
| --- | --- |
| Centricity RIS system | Age, sex, vital status and indication for CTAB, specialty of referring physician for both CTAB and DXA scan, DXA results and work-flow data |
| EPJ (EPIC) | Data osteoporosis diagnosis, DXA results |
| (SMR), which provides access to information on patients’ medication across Danish hospitals. | Data on medical treatment |

Table S6: The four different forms of exam done in the radiological department.

| **Exam** | **Description** |
| --- | --- |
| Acute exam | An exam addressing sudden, severe or urgent symptoms or conditions requiring rapid evaluation. |
| Diagnostic exam | An exam addressing non urgent symptoms of conditions, not requiring rapid evaluation. |
| Oncologic control exam | A follow-up exam with the purpose of monitoring the progression of cancer and effectiveness of treatments. |
| Other control scans | A follow-up exam with the purpose of monitoring the progression of disease (not cancer) and effectiveness of treatments. |

Table S7: Specialties of Referring physicians.

| Intensive Care Unit Emergency Department Surgical Department Medical Department Department of Dermatology General Practitioner Department of Oncology  Specialized Private Practitioner |
| --- |

Table S8: Specialties of the The Surgical and Medical department

| The Surgical Departments | Urology, Gastroenterology, Orthopedics, Ear, Nose and Throat, Gynecology, Dental-Oral Surgery, Vascular Surgery, Ophthalmology, Plastic surgery. |
| --- | --- |
| The Medical Department | Geriatric, Internal Medicine, Gastroenterology, Nephrology, Rheumatology, Cardiology, Infectious Medicine, Pulmonary Medicine, Neurology |

Table S9: CONSORT AI 2020 checklist

| CONSORT-AI 1a,b Elaboration | (i) Indicate that the intervention involves artificial intelligence/machine learning in the title and/or abstract and specify the type of model. | This is indicated in the title:  Opportunistic identification of vertebral compression fractures on CT scans of the chest and abdomen, using an AI algorithm, in a real-life setting. |
| --- | --- | --- |
|  | (ii) State the intended use of the AI intervention within the trial in the title and/or abstract. | This is stated in the abstract:  The HealthVCF was used to identify moderate and severe vertebral compression fractures (VCF) at a Danish hospital. |
| CONSORT-AI 2a (i) Extension | Explain the intended use of the AI intervention in the context of the clinical pathway, including its purpose and its intended users (e.g. healthcare professionals, patients, public). | This is explained in the introduction, **p 4 paragraph 2:**  The aim of our study was to assess the performance of an updated version of the algorithm in identifying prevalent moderate/severe VCFs on CT scans of thorax and abdomen (CTAB) in a real-life setting and to evaluate the impact of implementation on registration of diagnoses, referral to DXA and medical treatment. |
| CONSORT-AI 4a (i) Elaboration | State the inclusion and exclusion criteria at the level of participants. | This is stated in method section, **p. 6 paragraph 1:**  The study population includes both inpatients and outpatients at Zealand University Hospital in Koege referred for CTAB for all indications except fractures. The study included both women and men, aged 50 years and older. Individuals without a social security number (tourist) and individuals from other geographical regions were excluded. |
| CONSORT-AI 4a (ii) Extension | State the inclusion and exclusion criteria at the level of the input data. | This is stated in method section, **p. 6 paragraph 2:**  Exclusion criteria encompassed cases involving CTAB conducted as part of PET scans and instances where assessment of the vertebral column was not possible as judged by the radiographer/radiologist. CTAB for fracture identification were excluded, as well as cases of incomplete exams and wrong region of interest. |
| CONSORT-AI 4b Extension | Describe how the AI intervention was integrated into the trial setting, including any onsite or offsite requirements. | This is described in the methods section, **p. 4 paragraph 3:**  The HealthVCF, version 5.1.1, was installed and configured for the highest specificity on a server in January 2021 at the Department of Radiology, Zealand University Hospital (ZUH). It was set to identify Genant 2-3 (moderate/severe) VFCs on CTAB of patients referred to the Radiology Department during the study period, provided patients met the inclusion criteria. No human interaction was involved in handling of the input data.  If a moderate/severe VCF was identified by the HealthVCF, an additional scan image was added by the software to the CT scan series, before the examination was returned to the imaging system (Radiology Information system), RIS (Picture Archiving and Communication System, PACS). The HealthVCF used the Genant semi-quantitative method for diagnosing VCFs. The evaluation by the HealthVCF was done in real-time and did not delay the conduct of the CTAB. Scans identified with one or more moderate/severe compression fractures, were denoted positive scans. All scans evaluated by the HealthVCF were identified by accession number, registered on a weekly worklist with indications of the presence of moderate/severe VCF, and sent to the study team. |
| CONSORT-AI 5 (i) Extension | State which version of the AI algorithm was used. | This is described in the methods section, **p. 4 paragraph 3:**  The HealthVCF, version 5.1.1, was installed and configured for the highest specificity on a server in January 2021 at the Department of Radiology, Zealand University Hospital (ZUH). |
| CONSORT-AI 5 (ii) Extension | Describe how the input data were acquired and selected for the AI intervention. | This is described in the methods section, **p. 4 paragraph 3:**  It was set to identify Genant 2-3 (moderate/severe) VFCs on CTAB of patients referred to the Radiology Department during the study period, provided patients met the inclusion criteria. |
| CONSORT-AI 5 (iii) Extension | Describe how poor quality or unavailable input data were assessed and handled. | This is described in the methods section, **p. 6 paragraph 2:**  Exclusion criteria encompassed cases involving CTAB conducted as part of PET scans and instances where assessment of the vertebral column was not possible as judged by the radiographer/radiologist. CTAB for fracture identification were excluded, as well as cases of incomplete exams and wrong region of interest.. |
| CONSORT-AI 5 (iv) Extension. | Specify whether there was human-AI interaction in the handling of the input data, and what level of expertise was required of users. | This is specified in the method section, **p. 4 paragraph 3:**  No human interaction was involved in handling of the input data. |
| CONSORT-AI 5 (v) Extension | Specify the output of the AI intervention | This is specified in the methods section, **p. 4 paragraph 4:**  Scans identified with one or more moderate/severe compression fractures, were denoted positive scans. All scans evaluated by the HealthVCF were identified by accession number, registered on a weekly worklist with indications of the presence of moderate/severe VCF, and sent to the study team. |
| CONSORT-AI 5 (iv) Extension. | Explain how the AI intervention’s outputs contributed to decision-making or other elements of clinical practice. | This is explained in the methods section, **p. 5 paragraph 5:**  CTABs not flagged by the HealthVCF were not analyzed by the study-team but were analyzed by the radiologist at the time of CTAB, following standard procedure. For CTAB determined true positive for a VCF by the study team, the radiographer looked up the initial report. If no VCF had been reported, a standardized sentence was added to report alerting the referring physician to the VCF and noting that osteoporosis assessment was advised. If the VCF was already described in the initial report, no action was taken. |
| CONSORT-AI 19 Extension | Describe results of any analysis of performance errors and how errors were identified, where applicable. If no such analysis was planned or done, explain why not. | This is described in the methods section, **p. 6 paragraph 2:**  No performance error analysis was conducted as it was outside the study's scope. |
| CONSORT-AI 25 Extension. | State whether and how the AI intervention and/or its code can be accessed, including any restrictions to access or re-use. | This is stated in the declarations section, **p. 19 paragraph 3:**  The AI code is property of Nanox AI Ltd, and the authors had no access to the code during the study. |
